# Rapid Spread of SARS-CoV-2 in a State Prison After Introduction by Newly Transferred Incarcerated Persons — Wisconsin, August 14–October 22, 2020

**DOI:** 10.15585/mmwr.mm7013a4

**Published:** 2021-04-02

**Authors:** Rebecca B. Hershow, Hannah E. Segaloff, Abigail C. Shockey, Kelsey R. Florek, Sabrina K. Murphy, Weston DuBose, Tammy L. Schaeffer, Jo Anna Powell, MPH, Krystal Gayle, Lauren Lambert, Amee Schwitters, Kristie E.N. Clarke, Ryan Westergaard

**Affiliations:** ^1^CDC COVID-19 Response Team; ^2^Epidemic Intelligence Service, CDC; ^3^Wisconsin Department of Health Services; ^4^Wisconsin State Laboratory of Hygiene; ^5^Preventive Medicine Residency Program, Department of Population Health Sciences, University of Wisconsin School of Medicine and Public Health, Madison, Wisconsin; ^6^Department of Medicine, University of Wisconsin School of Medicine and Public Health, Madison, Wisconsin.

SARS-CoV-2, the virus that causes COVID-19, can spread rapidly in prisons and can be introduced by staff members and newly transferred incarcerated persons (*1*,*2*). On September 28, 2020, the Wisconsin Department of Health Services (DHS) contacted CDC to report a COVID-19 outbreak in a state prison (prison A). During October 6–20, a CDC team investigated the outbreak, which began with 12 cases detected from specimens collected during August 17–24 from incarcerated persons housed within the same unit, 10 of whom were transferred together on August 13 and under quarantine following prison intake procedures (intake quarantine). Potentially exposed persons within the unit began a 14-day group quarantine on August 25. However, quarantine was not restarted after quarantined persons were potentially exposed to incarcerated persons with COVID-19 who were moved to the unit. During the subsequent 8 weeks (August 14–October 22), 869 (79.4%) of 1,095 incarcerated persons and 69 (22.6%) of 305 staff members at prison A received positive test results for SARS-CoV-2. Whole genome sequencing (WGS) of specimens from 172 cases among incarcerated persons showed that all clustered in the same lineage; this finding, along with others, demonstrated that facility spread originated with the transferred cohort. To effectively implement a cohorted quarantine, which is a harm reduction strategy for correctional settings with limited space, CDC’s interim guidance recommendation is to serial test cohorts, restarting the 14-day quarantine period when a new case is identified (*3*). Implementing more effective intake quarantine procedures and available mitigation measures, including vaccination, among incarcerated persons is important to controlling transmission in prisons. Understanding and addressing the challenges faced by correctional facilities to implement medical isolation and quarantine can help reduce and prevent outbreaks.

## Investigation and Findings

Prison A is a medium-security state prison in Wisconsin with 300–350 staff members and a maximum capacity of 1,192 men housed in 15 units. Except for one unit (a restrictive housing unit with locked cells), all units have shared lavatories and common areas, including one 150-person dormitory-style unit with conjoined sleeping and common areas. Before the outbreak, prison A implemented multiple mitigation measures, including mandatory mask wearing for staff members and incarcerated persons.^†^ During August 17–19, 2020, members of a group of incarcerated men transferred from a Wisconsin central intake facility on August 13 were tested for SARS-CoV-2 by real-time reverse transcription–polymerase chain reaction (RT-PCR)^§^ in accordance with routine intake procedures^¶^; six received positive test results and were immediately isolated. Before testing, the new intake group was housed with other incarcerated persons, most of whom were recent transfers, in the intake unit. On August 24, testing of incarcerated persons in the intake unit identified six additional cases in incarcerated persons, who were immediately isolated. On August 25, intake processing was suspended, the intake unit was locked down,** and the remaining persons in the unit started a 14-day group quarantine. After receiving the test results from facilitywide testing on September 1, incarcerated persons with COVID-19 were moved to the intake unit, which potentially exposed quarantined persons to SARS-CoV-2. The 14-day quarantine that started on August 25 was not restarted after the potential exposure to persons with COVID-19. Mass (facilitywide) and targeted (selected units) testing was conducted on September 1, 14, and 23–24, and detected rapid spread; the percentage of positive test results among incarcerated persons was 2.4% on September 1 and increased to 46.2% on September 23.

After September 14 test results identified 86 cases of COVID-19 among incarcerated persons, the facility implemented a modified lockdown, restricting movement of incarcerated persons across units and shutting down common areas; however, staff members continued to rotate throughout the facility because of staffing shortages and scheduling policies. By September 22, the facility was unable to medically isolate or quarantine incarcerated persons because of limited space. On September 28, the Wisconsin DHS and Wisconsin Department of Corrections contacted CDC to request assistance in investigating the outbreak.

A COVID-19 case was defined as a positive SARS-CoV-2 test result^††^ received by any incarcerated person or staff member at prison A during August 14–October 22, 2020. Voluntary testing was offered to incarcerated persons during mass or targeted testing, routine intake screening (on postintake days 4 or 5), or when symptoms were reported. Staff members were tested at the first two mass testing events and were instructed to report receipt of positive test results from outside testing. Epidemiologic data were extracted from prison-managed documents and the Wisconsin Electronic Disease Surveillance System. Attack rates were calculated using population estimates communicated by prison A. A heat map was created to show the percentage of new cases across units and testing events, assuming maximum capacity. This activity was reviewed by CDC and was conducted consistent with applicable federal law and CDC policy.^§§^

**FIGURE 1:**
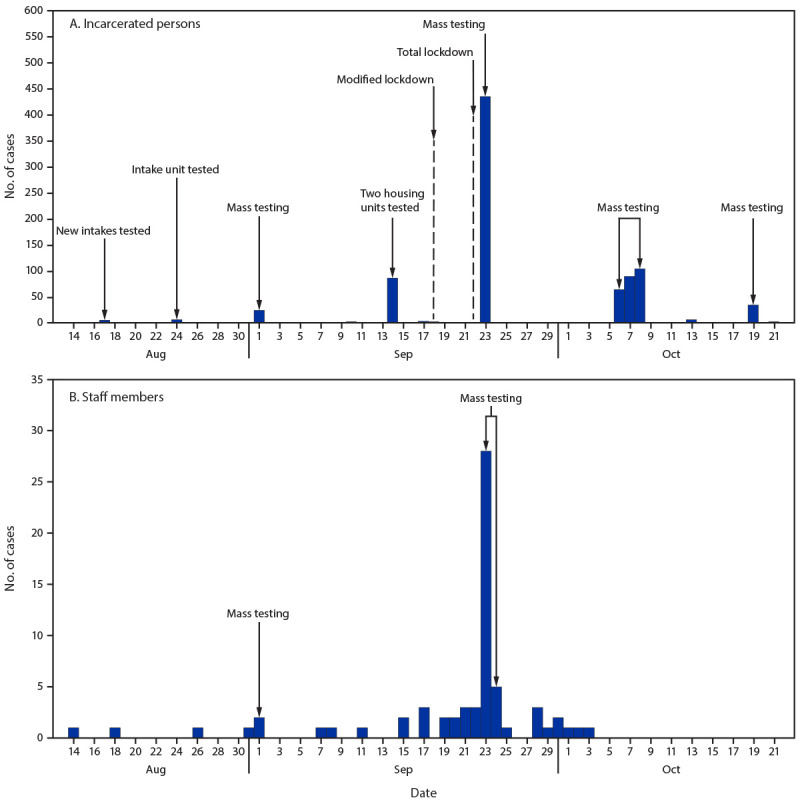
Number of COVID-19 cases among incarcerated persons (A) (n = 869) and staff members (B) (n = 69), by testing date — prison A, Wisconsin, August 14–October 22, 2020* * Modified lockdown refers to prison A’s policy change that restricted movement of incarcerated persons within the facility and shut down all areas except for food services. Total lockdown refers to prison A’s policy change that restricted outdoor recreation and limited movement within housing units by modifying daily operations to allow incarcerated persons to leave their cells only in small groups during assigned time slots for shower and telephone time. Meals were delivered and eaten within cells.

On September 1, a total of 8 days after cases were identified on the intake unit, facilitywide testing identified cases in six of 15 units (Supplementary Table, https://stacks.cdc.gov/view/cdc/104507). Twenty-two days later (September 23), cases were identified across all 15 units, with infections in three units progressing from zero cases to attack rates ranging from 66.2% to 84.6% during that period. During August 14–October 22, a total of 869 (79.4%) of 1,095 incarcerated persons (median age = 36 years, range = 18–83 years) and 69 (22.6%) of 305 staff members (median age = 44 years, range = 23–77 years) received positive SARS-CoV-2 test results (Figure 1). Among 869 incarcerated persons with COVID-19, 118 (14%) were infected in the dormitory-style unit (unit 15). Six incarcerated persons were hospitalized (median age = 58 years, range = 33–69 years), one of whom, a man aged 56 years, died. Mass or targeted testing identified 95.4% (829 of 869) of cases in incarcerated persons and 42.0% (29 of 69) of cases in staff members. In the 14 days before reporting onset of COVID-19 symptoms or receiving positive SARS-CoV-2 test results, 71 (8.2%) incarcerated persons transferred units, and 27 (39.1%) staff members were assigned to more than one unit.

## Whole Genome Sequencing

All 409 of 869 (47.1%) nasal swab specimens from incarcerated persons sent to the Wisconsin State Laboratory of Hygiene for testing were retained for WGS, and 172 (42%) of these, representing 20% of cases among incarcerated persons across 13 of 15 units, were successfully sequenced. These included specimens from cases identified from intake testing (12 of 12), symptomatic testing (11 of 22), and mass or targeted testing on September 14 (66 of 86), October 6–7 (60 of 153), and October 19 (23 of 34). No specimens from staff members were available for sequencing because the testing laboratory had discarded them.

**FIGURE 2:**
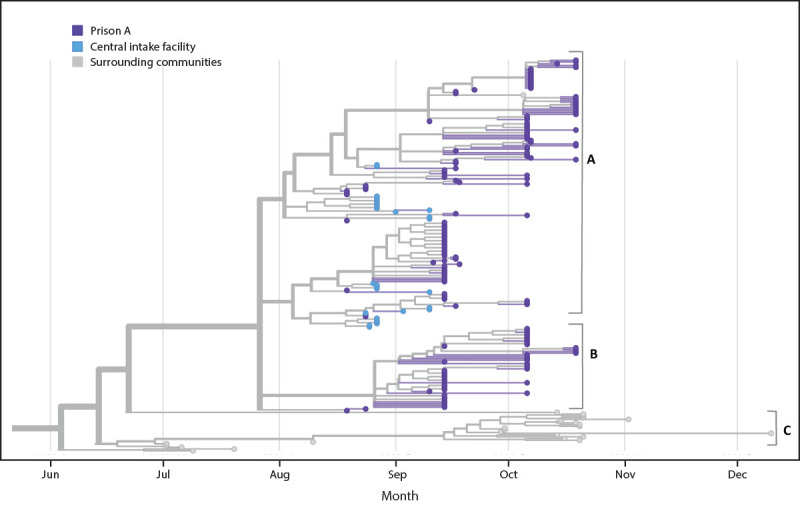
Phylogenetic tree* showing genetic distance between available SARS-CoV-2 specimens^†^ from prison A,^§^ a central intake facility, and the surrounding communities — Wisconsin, June–December 2020 * Includes 230 of 1,345 sequences produced by Wisconsin State Laboratory of Hygiene during June–December 2020 using the ARTIC sequencing approach (https://artic.network/ncov-2019) on both the Illumina MiSeq and Oxford Nanopore MinION platforms. Consensus sequences generated using the StaPH-B ToolKit Monroe workflow v1.2.5 (https://github.com/StaPH-B/staphb_toolkit). Phylogenetic inference and visualization performed using Nextstrain Augur v9.0.0 (https://github.com/nextstrain/augur) and Nextstrain Auspice v2.18.4 (https://github.com/nextstrain/auspice). ^†^ Clusters A and B refer to sequences from specimens collected at prison A. Cluster A refers to sequences from specimens collected at prison A that showed a genetic relationship with 29 sequences collected from a concurrent outbreak at the central intake facility. Cluster C refers to sequences from specimens collected outside of the outbreaks at the central intake facility and at prison A. ^§^ No specimens from staff members were available for sequencing because the testing laboratory had discarded them.

Sequences were compared with those from specimens obtained from persons in the central intake facility, the location of persons with the initial cases before their transfer to prison A, and others across Wisconsin (Figure 2). Sequences from specimens collected at prison A showed a genetic relationship with 29 sequences collected from a concurrent outbreak in the central intake facility (cluster A). Specimens from prison A (clusters A and B) were more closely related to each other than to other sequences outside of these outbreaks (cluster C).

## Public Health Response

On October 16, CDC and Wisconsin DHS recommended that prison A house incarcerated persons with active infection separately from susceptible incarcerated persons^¶¶^ and avoid housing susceptible incarcerated persons in the dormitory-style unit. Prison A began immediately implementing these recommendations. The facility was advised to retest quarantined cohorts every 3–7 days and restart the 14-day quarantine period whenever a new case was identified. However, testing capacity was insufficient to implement serial testing of quarantined cohorts. Case counts decreased substantially after the investigation period; no cases were reported after January 15, 2021.

## Discussion

Investigation findings demonstrate that SARS-CoV-2 spread rapidly in prison A, infecting 79% of incarcerated persons and 23% of staff members in 2 months. Factors that likely facilitated transmission include the described intake quarantine procedures, crowded housing units, and movement of incarcerated persons and staff members among units.

WGS detected one cluster of cases originating in a group of newly transferred incarcerated persons, with all subsequent cases clustering closely together. CDC’s interim guidance recommendation is to quarantine incarcerated persons at intake and immediately isolate symptomatic persons and persons who receive positive test results (*3*). If incarcerated persons are quarantined in a group, retesting the cohort is recommended every 3–7 days; if any person in the cohort receives a positive test result, the 14-day quarantine period should restart for the remaining cohort (*4*).

Incarcerated persons quarantined at intake were tested only on days 4 or 5 after entry because of resource and staffing constraints. Antigen testing offers a feasible option for repeat testing of persons in cohorted quarantine or during an outbreak, when widespread use of nucleic acid amplification tests is infeasible; however, confirmation from such tests might be needed (e.g., for asymptomatic persons with a known exposure or symptomatic persons who receive negative antigen test results) (*5*).

Because of ongoing within-facility movement of staff members and incarcerated persons, staff members and incarcerated persons both might have contributed to the prolonged outbreak. Assignment of staff members to specific units and routine testing of staff members might reduce within-facility and community transmission (*1*,*6*,*7*); however, sufficient staffing and testing resources would be needed. Other prevention measures such as movement restriction during an outbreak should be considered for staff members and incarcerated persons. Prioritization of vaccination of incarcerated persons and staff members could play an important role in preventing outbreaks in prisons (*8*).

The findings in this report are subject to at least four limitations. First, sociodemographic or clinical comparisons between persons who did and did not have COVID-19 could not be made because data were available only for infected persons. Second, cases in staff members were likely underreported because facilitywide testing and test result reporting were not mandated. Third, maximum capacity was used to calculate the percentage of new cases detected by unit because the number of persons in each unit over time was unavailable. Finally, only 20% of specimens from incarcerated persons with COVID-19 and no specimens from staff cases were sequenced, limiting understanding of staff member and prison-to-community transmission.

SARS-CoV-2 transmission is difficult to control in congregate living facilities (*2*,*9*). Investigation findings highlight challenges in implementing quarantine guidance and structural factors that contribute to rapid spread, including limited testing resources and space for quarantine and medical isolation. Adjustments to prison capacity, organization, and intake and quarantine processes might be necessary to protect staff members and incarcerated persons from COVID-19 outbreaks and to prevent community transmission (*10*). This investigation demonstrates the importance of identifying and addressing barriers to adherence to public health guidance on COVID-19 management in correctional settings. In addition, because vaccination of staff members alone likely would have been insufficient to prevent the outbreak described in this report, incarcerated persons and correctional facility staff members should both be vaccinated as early as possible to help prevent outbreaks and the associated morbidity and mortality of incarcerated persons and staff members (*8*).

**Summary**

**What is already known about this topic?**

SARS-CoV-2 can spread rapidly in prisons and can be introduced by staff members and newly transferred persons.

**What is added by this report?**

After early detection of SARS-CoV-2 in six newly transferred persons during intake quarantine in a Wisconsin prison, 79.4% of incarcerated persons and 22.6% of staff members contracted SARS-CoV-2 during August 14–October 22, 2020. Whole genome sequencing from 172 incarcerated persons with COVID-19 determined that all specimens clustered in the same lineage.

**What are the implications for public health practice?**

Insufficient quarantine after intake can lead to rapid, widespread SARS-CoV-2 transmission, even after early detection of initial cases. Understanding and addressing the challenges faced by correctional facilities to implement medical isolation and quarantine can help reduce and prevent outbreaks.

## Footnotes

* These authors contributed equally to this report.

† As of July 2020, the following mitigation measures were implemented at prison A: 1) mandatory mask wearing for staff members and incarcerated persons when outside their cells or away from their sleeping areas; 2) prevention of large gatherings by limiting the number of incarcerated persons who could congregate in common recreation areas and modifying schooling and other programming sessions; 3) preventive or wellness screenings led by health services staff members (daily for incarcerated persons in quarantine and semiroutinely for other incarcerated persons across the facility to identify susceptible incarcerated persons who were not actively reporting symptoms to their unit officers); 4) symptom screening and temperature checks of staff members and other essential visitors on entry into the facility; 5) suspension of in-person visits to incarcerated persons; and 6) modification of daily operations to reduce close contact between incarcerated persons within the intake unit.

§
https://www.fda.gov/media/137450/download

¶ Five of the six newly transferred incarcerated persons with COVID-19 were tested on August 17 (postintake day 4), and one was tested on August 19 (postintake day 6) after initially refusing testing on August 17. Routine intake procedures dictate that newly transferred incarcerated persons are tested on postintake days 4 or 5.

** Movement of incarcerated persons into or out of the unit was restricted. All incarcerated persons under quarantine within the unit were isolated in their cells except for telephone calls, meals, and bathroom or shower use.

†† All cases were identified with laboratory-confirmed RT-PCR testing, except three cases among staff members who received antigen tests elsewhere.

§§ 45 C.F.R. part 46; 21 C.F.R. part 56; 42 U.S.C. Sect. 241(d), 5 U.S.C. Sect. 552a, 44 U.S.C. Sect. 3501 et seq.

¶¶ Susceptible persons were defined as those who had never received a positive SARS-CoV-2 test result or whose date of infection onset was at least 90 days earlier. https://www.cdc.gov/coronavirus/2019-ncov/hcp/duration-isolation.html
